# Solution characterization of the dynamic conjugative entry exclusion protein TraG

**DOI:** 10.1063/4.0000171

**Published:** 2022-12-27

**Authors:** Nicholas Bragagnolo, Gerald F. Audette

**Affiliations:** Centre for Research on Biomolecular Interactions, Department of Chemistry, York University, Toronto, Ontario M3J 1P3, Canada

## Abstract

The R100 plasmid and the secretion system it encodes are representative of F-like conjugative type IV secretion systems for the transmission of mobile DNA elements in gram-negative bacteria, serving as a major contributor to the spread of antibiotic resistance in bacterial pathogens. The TraG protein of F-like systems consists of a membrane-bound N-terminal domain and a periplasmic C-terminal domain, denoted TraG*. TraG* is essential in preventing redundant DNA transfer through a process termed entry exclusion. In the donor cell, it interacts with TraN to facilitate mating pair stabilization; however, if a mating pore forms between bacteria with identical plasmids, TraG* interacts with its cognate TraS in the inner membrane of the recipient bacterium to prevent redundant donor–donor conjugation. Structural studies of TraG* from the R100 plasmid have revealed the presence of a dynamic region between the N- and C-terminal domains of TraG. Thermofluor, circular dichroism, collision-induced unfolding–mass spectrometry, and size exclusion chromatography linked to multiangle light scattering and small angle x-ray scattering experiments indicated an N-terminal truncation mutant displayed higher stability and less disordered content relative to full-length TraG*. The 45 N-terminal residues of TraG* are hypothesized to serve as part of a flexible linker between the two independently functioning domains.

## I. INTRODUCTION

Horizontal gene transfer (HGT) methods, such as transformation of extracellular DNA, transduction of viral particles, and conjugation, are utilized by bacteria to acquire and integrate novel genes into their host chromosome through homologous recombination; if these genes provide the bacteria a selective advantage, HGT often increases the rate of dissemination of these virulence genes.^1–4^ In this way, HGT enhances the rate of bacterial evolution by increasing the plasticity of the bacterial genome.^1,5–7^ Extrachromosomal DNA, such as plasmids, can have broad species-host ranges and do not require gene transposition events for proper gene function, thus amplifying the propagation of virulence genes in bacterial communities.^8–10^ Conjugation is enabled by a type IV secretion system (T4SS) transcribed and translated from the transfer (*tra*) or virulence (*vir*) gene region of a conjugative plasmid.^10,11^ As genes enhancing bacterial virulence, including antibiotic resistance genes, can integrate into conjugative plasmids, many of which have broad-host ranges, conjugation is considered to be the most prominent contributor to the spread of virulence genes in bacterial pathogens.^12–14^

**FIG. 1.**
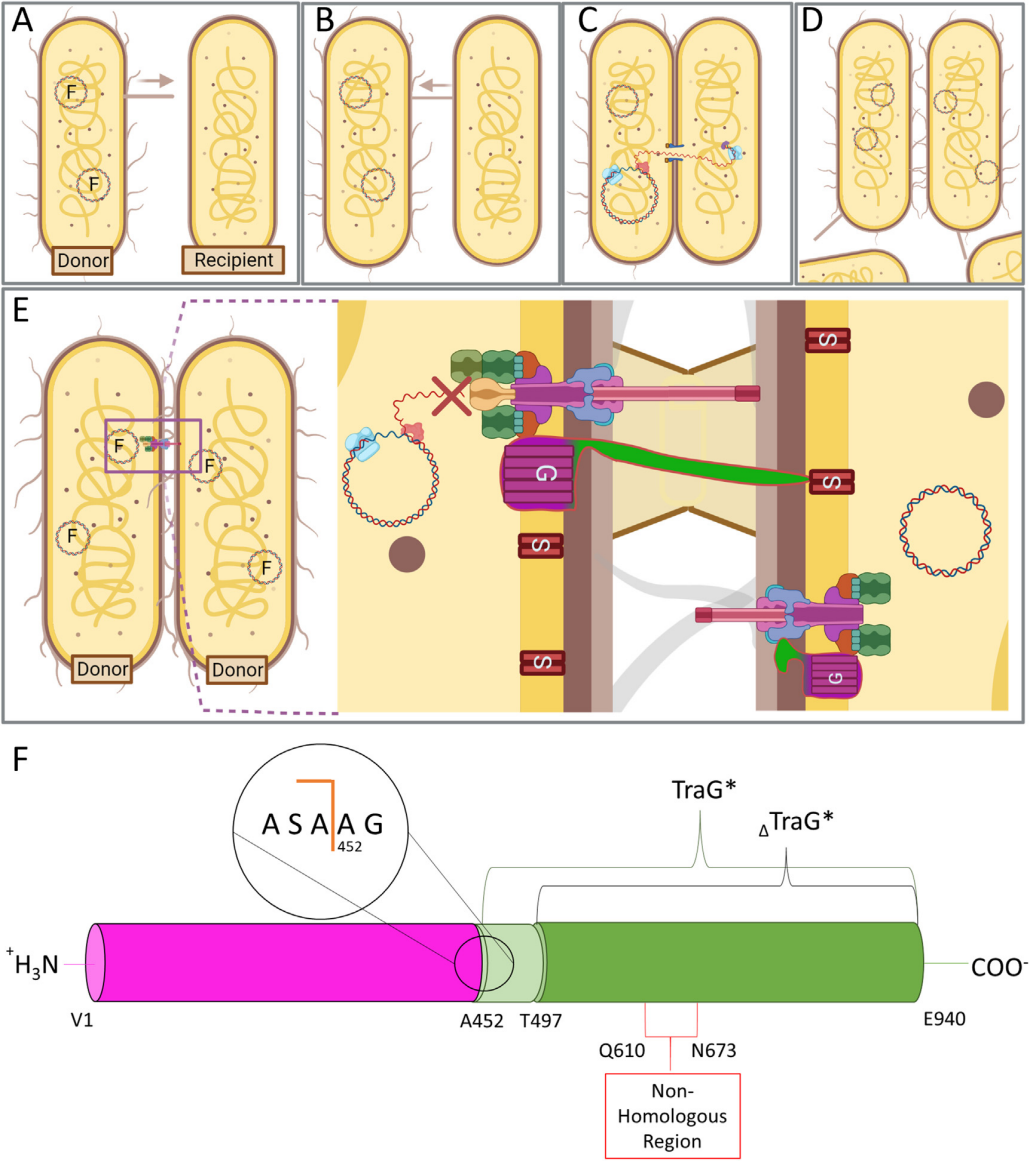
A simplified depiction of gram-negative bacterial conjugation and entry exclusion by TraG in F-like T4SSs. (a) Donor bacteria possessing F-like plasmids coding for conjugative T4SS are piliated and will repeatedly extend and retract conjugative pili to contact a neighboring bacterium.^101^ (b) Pilus retraction occurs when a neighboring recipient bacterium's cell wall has been contacted by the pilus tip of the donor cell, allowing for adhesion.^102^ (c) Donor and recipient bacteria are brought into close proximity, and after surface and entry exclusion steps, a mating junction forms resulting in the fusion of lipid bilayers. The synchronous transfer and replication of a single strand of nicked plasmid DNA from the donor to recipient is then mediated by the relaxasome (pink) and transferosome (purple).^6^ Rolling circle replication by DNA replication machinery (light blue) in the recipient allows for synthesis of the complementary strand of the plasmid DNA. (d) The conjugative transfer event ends after the single-stranded DNA is transferred to the recipient cell and the mating junction has closed. The recipient cell, following synthesis of the complementary strand of the transferred plasmid DNA, is now a donor cell for the conjugative plasmid. (e) In a colony of bacteria carrying plasmids with identical transfer (*tra*) regions, conjugation between donor cells is prevented through entry exclusion (Eex), proposedly in the event of faulty surface exclusion.^32,100^ The formation of a complete mating pore and the transfer of redundant plasmid DNA is prevented through interaction of TraG in the donor cell with a cognate TraS embedded in the recipient's IM. [Figures (a)–(e) made with Biorender]. (f) Generalized domain organization of TraG. The N-terminal transmembrane region of the protein responsible for aiding in pilus assembly is colored in magenta and the C-terminal periplasmic region termed TraG* is colored in green. These regions are separated by a signal I peptidase cleavage site at residue A451.^32^ The 45-residue truncation mutant construct is labeled as _Δ_TraG* and contains the region responsible for interaction with TraS. The non-homologous region, which differs between TraG_F_ and TraG_R100_, is responsible for Eex. The region shaded in light green from A452 to A496 represents a flexible linker region between the two domains.

In conjugation, plasmids or chromosomally integrated conjugative elements (ICEs) are replicated in a donor cell and are transferred into a recipient cell in a donor-controlled fashion using a T4SS.^3–6,15^ T4SSs are the most ubiquitous secretion system in prokaryotes; natural selection has favored conjugation as donor-mediated HGT appears to be a necessity for survival based on the requirement for rapid evolution in the competitive environments that unicellular organisms find themselves in.^6,7,11,16^ The role of the T4SS is (a) to process and secrete self-polymerizing pilin monomers to form a pilus that will extend to contact a neighboring recipient cell and (b) depolymerize at the base to retract the pilus to bring the cells in proximity, allowing for cell membrane fusion and (c) DNA transfer (Fig. 1). The T4SS is a large and dynamic multi-protein complex; in gram negative bacteria, it spans both inner (IM) and outer membranes (OMs).^16–19^ The F T4SS is the representative member of a subset of gram negative bacterial T4SS known as the thick flexible T4SS complexes, and the F plasmid which encodes it is the best characterized conjugative plasmid in gram negative bacteria.^6,17,20^ The F plasmid was the first conjugative plasmid discovered, as characterized by Lederburg and Tatum and termed the “Fertility” or “F” factor.^21^ F and F-like plasmids are unique in their host range as they are most commonly found in gram negative, facultative anaerobic bacterial genera such as *Klebsiella*, *Salmonella*, and *Escherichia*.^22^ These species can survive harsh conditions and are common human pathogens. The R100 plasmid is from the F-like plasmid family based on genetic phylogeny to relaxase and coupling protein genes, and differs from F at the level of relaxosome formation, regulation, pilus serology, pilus-specific phage sensitivity, surface and entry exclusion between the two transfer systems, and in replicon type.^23–25^ The R100 plasmid is one of the classic F-like plasmids; it was determined to be widespread among gram negative bacterial species due to its high propensity to harbor antibiotic resistance genes.^22,26^ These systems are important to study as increasing numbers of multi-drug resistant bacterial pathogens have been observed globally as attributed to poor nosocomial hygiene, excessive use of antibiotics in animal agriculture, over-prescription of broad spectrum antibiotics, and furthered by the COVID-19 pandemic due to de-prioritization of bacterial infection prevention and control, over-sanitization with biocides, shortage of personal protective equipment, and crowding of infected hospital patients.^27–31^

In the F-like T4SS superstructure, two IM-bound proteins TraG and TraS are responsible for an event known as entry exclusion (Eex), which functions to prevent redundant donor–donor DNA transfer [Fig. 1(e)].^32^ TraG in the donor cell is thought to scan the IM of the recipient cell and interact with the cognate TraS to prevent conjugation. If the TraG–TraS interaction does not occur then TraG interacts with TraN in the OM of the donor cell, resulting in mating pair stabilization (Mps) and advancing the conjugation process.^17,33^ Interestingly, Eex systems of the closely related plasmids F and R100 retain plasmid specificity; TraG of the T4SS transfer apparatus present in donor cells will interact with the cognate TraS in the recipient cell to prevent redundant conjugation, but will not interact with TraS from a different plasmid and therefore allow conjugative DNA transfer.^32^ Additionally, TraG will not interact with TraS in the same IM, and it functions in a trans fashion only.

TraG is a ∼103 kDa protein with an N-terminal polytopic IM-bound domain and a periplasmic C-terminal domain from amino acid residues A452-Q940 referred to as TraG* [Fig. 1(f)].^32,34^ TraG is multifunctional as the N-terminal domain is responsible for pilus assembly, and the periplasmic TraG* is required for Mps and Eex; the protein must be intact for proper function in both pilus assembly and Eex/Mps.^32,35,36^ TraG from the F (TraG_F_) and R100 (TraG_R100_) plasmids have an overall sequence identity of 93%; however, the region in TraG* from residues 610 to 673 shows only 17%. This region of TraG was shown to be responsible for plasmid specificity in entry exclusion, indicating the role of the region for interacting with their cognate TraS.^32^

A high-resolution structure of TraG* has the potential to elucidate the mechanism by which TraG interacts with the cognate TraS of a recipient cell to affect Eex. Disrupting the Eex process to promote lethal zygosis through ceaseless conjugation, or to activate TraG and the Eex process to prohibit conjugation, could allow for the development of a novel class of antibiotics.^37,38^ In this study, we report on the structural dynamicity of TraG* in solution. The molecular weight of TraG* (∼50 kDa) complicates nuclear magnetic resonance studies, and the structural dynamicity of the protein complicates crystallization. Protein modeling and analysis using the phase separating protein predictor (PSP)^39^ software identified a highly flexible N-terminal domain that is hypothesized to serve as a linker region between the two functional TraG domains, which is supported with predicted models from RoseTTAFold and AlphaFold.^39–42^ An N-terminal truncation of TraG* from the R100 plasmid (TraG*_R100_) was designed based on these data, henceforth referred to as _Δ_TraG*_R100_. The thermal, chemical, and conformational stability of this mutant was confirmed to be improved relative to TraG*_R100_ using differential scanning fluorimetry (also referred to as Thermofluor assays), circular dichroism (CD), and collision induced unfolding–mass spectrometry (CIU–MS). Size exclusion chromatography linked to multiangle light scattering and small angle x-ray scattering (SEC–MALS–SAXS) studies shows the improved solubility of _Δ_TraG*_R100_ based on a lowered propensity for aggregation resulting in improved data and a more reliable bead model reconstruction.

## II. MATERIALS AND METHODS

Details into the expression and purification of TraG* proteins in this study can be found in Sec. S1 of the supplementary material.^105^

### A. Differential scanning fluorimetry/Thermofluor assay

To quantify the beneficial properties of buffers and salts in aiding the stability of proteins, and to compare the thermal stability of TraG*_R100_ and _Δ_TraG*_R100_, Thermofluor assays were performed. Thermofluor involves the use of a fluorescent hydrophobic probe to determine optimal solvent-protein interactions using the melting temperature (T_m_) of the protein as an indicator of stability.^43–46^ SYPRO orange dye was used as the probe, and it was prepared through serial dilution from a 5000× commercial stock (ThermoFisher) to a final concentration of 10× in the buffers of interest, with 1 mg/ml of TraG*_R100_ (purified with 5% glycerol) in a total of 50 *μ*l. Fluorescence output was measured on a RotorGeneQ thermocycler (Qiagen) with output set to 470 nm and detected at 610 nm, with a gain of 7; samples were heated from 25 to 99 °C at a ramp rate of 1 °C/min. The choice of buffers used for this assay was inspired by Seabrook and Newman.^46^ A total of 14 buffers were tested at 50 mM concentration with either 50 mM NaCl or 200 mM NaCl, and each condition was performed in triplicate, with a lysozyme positive control in 1 × PBS (pH 7.4), dye-only negative control and a protein-only negative control in every experiment. This was repeated for both proteins to gather a total of six data sets for each buffer condition. The data were analyzed using DSFworld^47^ which allowed for crude normalization, averaging of the curves to produce more accurate T_m_ values and aided in creating graphs of the output thermal melting curves.

### B. Circular dichroism

The molar ellipticity (mdeg) of TraG*_R100_ mutants was measured by CD spectroscopy at a protein concentration of 2 *μ*M over wavelengths 190–260 nm. To normalize the CD spectra of the proteins, the spectrum of the solvent [10 mM 2-(N-morpholino)ethanesulfonic acid (MES) pH 6.5, 10% glycerol] was subtracted from the experimental spectra. All spectra were obtained on a J-815 CD spectrometer (Jasco) through a continuous scan performed at 100 *μ*m/min with molar ellipticity measurements every 0.1 nm, with an accumulation factor of 8. Urea denaturation experiments were performed to provide an indication of protein chemical stability when comparing TraG*_R100_ with and without the putative intrinsically disordered region (IDR), as denaturing studies are common for determining the relative stability of protein mutants.^48–50^ TraG*_R100_ variants were added to solutions with different urea concentrations such that the final concentration of protein was 2 *μ*M. Samples were incubated at 25 °C for 1 h prior to CD measurements. Urea concentrations of 0.5, 1, 2, 3, 4, and 5 M to determine the approximate range for protein denaturation. Spectra measurements were performed with the same scanning protocol as stated above, and all results were normalized to their respective solvent conditions. Deconvolution of all CD data was performed using BeStSel,^51^ an algorithm for protein fold recognition and secondary structural determination using input CD spectra, unique in its ability to distinguish parallel from antiparallel β-sheets and useful in converting raw data measurements of molar ellipticity to Δε based on concentration, molecular weight of the protein, and path length (0.1 cm).

### C. Collision induced unfolding mass spectrometry

TraG*_R100_ samples were prepared for MS analysis using a 5 kDa molecular weight cutoff (MWCO) dialysis cassette to buffer exchange the protein at 4 °C into 100 mM ammonium acetate (MS grade), pH 6.6. Native MS analysis was performed on a Synapt G2S (Waters) with 5 *μ*M TraG*_R100_ or _Δ_TraG*_R100_ flowing at a rate of 5 *μ*l/min. In the collision induced unfolding (CIU)–MS experiments, data were collected from 5 V trap collision energy (CE) to 150 V in 5 V increments, with an acquired m/z range of 2000–5000 with no manual trapping. Capillary voltage was set to 3.0 kV, the sampling cone was 150.0, with source and desolvation temperatures at 120 and 250 °C, respectively, cone gas flow 71.0 l/h, nanoflow gas pressure at 2 bar, desolvation gas flow at 600 l/h, the transfer CE was 10.0 V, the trap gas flow was 4.0 ml/min, and the ion mobility spectrometry (IMS) wave delay was 1 ms with a wave height start of 10 V and a wave end height of 40 V.

### D. SEC–MALS–SAXS

TraG*_R100_ and _Δ_TraG*_R100_ were purified as described (supplementary materials Sec. S1^105^); however, a desalting step was not performed. The protein was dialyzed using a 25 kDa MWCO dialysis membrane into 20 mM 4-(2-hydroxyethyl)-1-piperazineethanesulfonic acid (HEPES), 100 mM NaCl, 5% glycerol, (and 0.05% NP40 for TraG*_R100_) pH 7.0, and further dialyzed into 30 kDa MWCO concentrator using a 10× dilution of a buffer stock [200 mM HEPES, 1 M NaCl, 50% glycerol (0.5% NP40 for HisTraG*_R100_), pH 7.0, 0.2 *μ*m membrane filtered]. Three hundred milliliters of this buffer stock (200 ml for the NP40 buffer) was chilled at −20 °C and sent to the BioCAT facility at the Advanced Photon Source along with 1000 *μ*l of 5 mg/ml HisTraG*_R100_, and 1000 *μ*l of 5 mg/ml His_Δ_TraG*_R100_, as quantified using the Edelhoch method. SAXS diffraction data were collected using the parameters seen in Table I. Samples were injected onto a Superdex 200 10/300 Increase SEC column (Cytiva) using an Agilent Infinity II HPLC, then sent through a Wyatt DAWN Heleos II MALS instrument and a Wyatt Optilab T-rEX direct refractive index (dRI) detector, and finally into the SAXS flow cell in the path of the synchrotron beamline. Data were analyzed using the RAW and ATSAS software packages.^52,53^ Bead models were generated from the output files of 20 cycles using the integrated DAMMIF tool with DAMAVER averaging and refinement, and clustering using DAMCLUST. SAXS data for TraG* from the R100 plasmid was submitted to SASBDB (https://www.sasbdb.org) under accession codes SASDQG6 (ΔTraG*_R100_) and SASDQH6 (TraG*_R100_).^104^

**TABLE I.** SEC–MALS–SAXS data collection parameters for HisTraG*_R100_, and His_Δ_TraG*_R100_.

Data collection parameters		
Instrument	BioCAT (Sector 18, APS)	
Detector	Eiger2 XE 9M	
Wavelength (Å)	1.033	
q-measurement range (1/Å)	0.0028–0.42	
Exposure time (s)	0.5	
Size exclusion column	Superdex 200 10/300 increase	
Flow rate (ml/min)	0.6	
Temperature (°C)	20	
Protein	HisTraG*_R100_	His_Δ_TraG*_R100_
Concentration (mg/ml)	5.0	4.8
Loaded volume (*μ*l)	300	300
Buffer	20 mM HEPES, 100 mM NaCl, 5% glycerol, 0.05% NP40, pH 7.0	20 mM HEPES, 100 mM NaCl, 5% glycerol, pH 7.0
Structural parameters		
*I*(0) (cm^−1^) [from P(r)]	0.79 ± 9.46 × 10^−3^	0.02 ± 3.9 × 10^−5^
R_g_ (Å) [from P(r)]	91.23 ± 3.4	44.14 ± 0.23
*I*(0) (cm^−1^) (from Guinier)	0.71 ± 4.13 × 10^−3^	0.02 ± 3.11 × 10^−5^
R_g_ (Å) (from Guinier)	57.68 ± 0.73	41.27 ± 0.81
D_max_ (Å)	450	175

## III. RESULTS

### A. Structural prediction software demonstrated large loop regions in TraG models

**FIG. 2.**
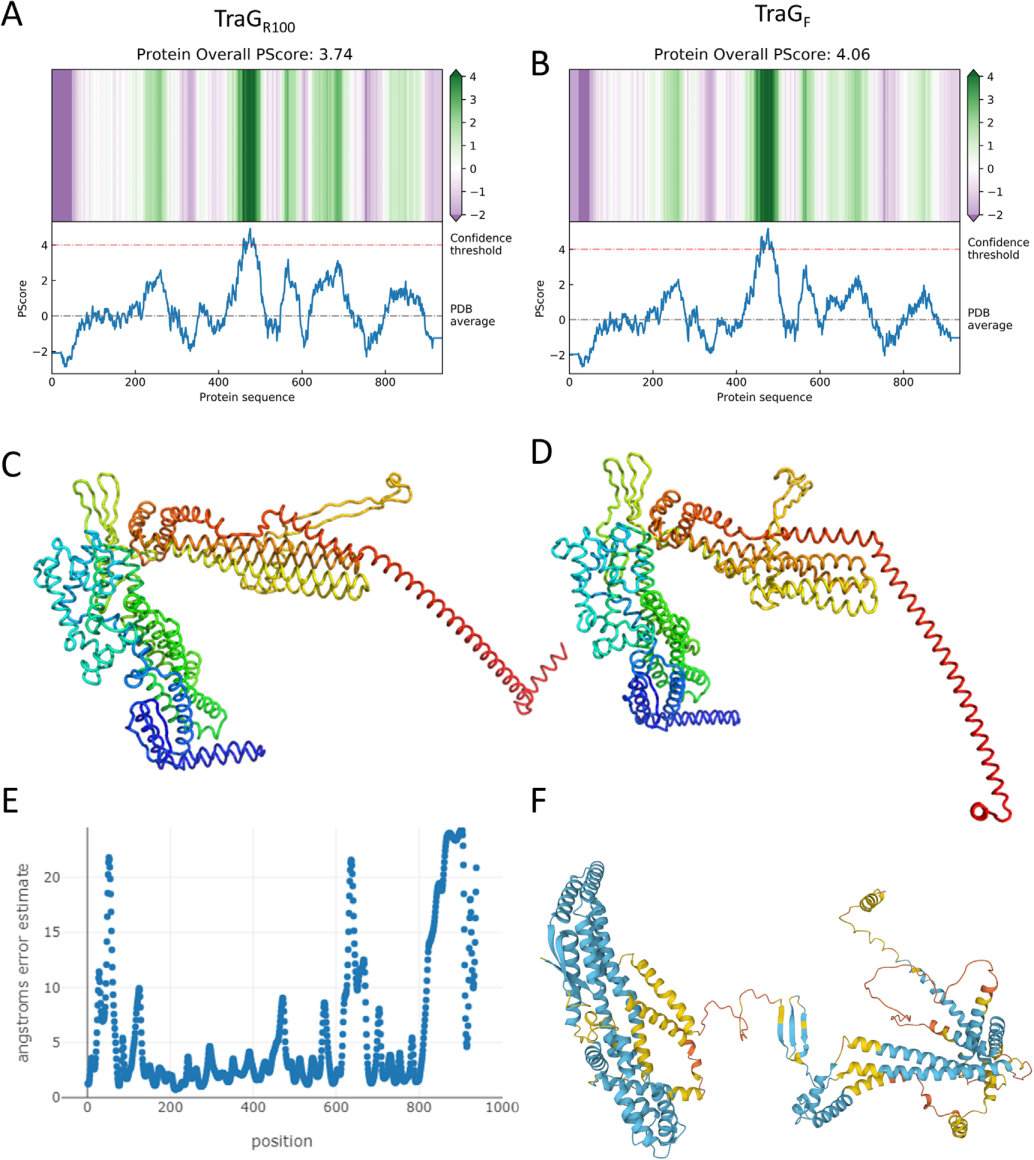
Predicted disorder content and structural models of TraG constructs from F and R100 plasmids using the PSP, RoseTTAfold and AlphaFold.^39–42^ The upper images show the resultant 2D heat maps and graphs produced by entering the (a) TraG_R100_ and (b) TraG_F_ sequence into the PSP.^39^ The only region predicted to be a phase separating IDR is the putative linker from residues 447 to 498, as it is the only region that surpasses the designated confidence threshold. (c) TraG_R100_ as predicted by RoseTTAfold, color mapped by chainbow from N- to C- terminus, with a confidence value of 0.55. (d) TraG_F_ as predicted by RoseTTAfold, with a confidence value of 0.52; the corresponding chart displaying error estimate per modeled residue is shown in (e). (f) Full-length TraG_F_ as predicted by AlphaFold, color mapped based on per residue modeling confidence, where blue is 90%–70%, yellow is 70%–50%, and orange is <50%. No residue in the model is mapped with high confidence (>90%). RoseTTAfold models were displayed using Pymol.^103^

The phase separation predictor (PSP) software for predicting intrinsically disordered regions (IDRs), which relies upon the propensity of a primary protein sequence for long range pi–pi contacts,^39^ provided insight in determining which regions of TraG, the conjugative entry exclusion protein found in F-like T4SS, should be targeted for mutation or deletion to improve protein stability. The region from residues 447–498 is predicted to have phase separating qualities in both TraG from the F plasmid (TraG_F_) and TraG from the R100 plasmid (TraG_R100_), with 27 of these residues surpassing the PSP algorithm's score threshold for the designation of residues as intrinsically disordered (4.0),^39^ thus providing TraG_F_ and TraG_R100_ with overall scores of 4.06 and 3.74, respectively [Figs. 2(a) and 2(b)]. The lower score of TraG_R100_ deemed it more favorable to undertake further experiments.

The predicted model of TraG_F_ in the AlphaFold database (code: AF-B1VCA9-F1) indicated high–medium confidence in the prediction of the N-terminal membrane bound domain with medium-low confidence throughout the C-terminal periplasmic domain [Fig. 2(f)].^40,41^ Notably, the hypothesized flexible linker region from A452 to A496 and the non-homologous region from R610 to D673 were predicted to be loop regions in the RoseTTAFold [Fig. 2(d)] and AlphaFold [Fig. 2(f)] models. However, a portion of the putative linker region was modeled as a pseudo β-sheet in RoseTTAFold and a β-sheet in AlphaFold. In all models, the final 100 residues of the protein are poorly predicted; the plot displaying the per-residue error estimate in Fig. 2(e) illustrates these observations. Similar predictions were made in the TraG_R100_ RoseTTAFold models; however, the confidence value of the model was slightly higher at 0.55 vs TraG_F_ at 0.52. The modeling software provided some distinctions in the TraG models, in that the C-terminus is largely unstructured in the AlphaFold TraG_F_ model, while using RoseTTAFold it is predicted to be a large helix modeled in different orientations depending on *in silico* N-terminal deletions and sequence changes (supplementary material Fig. S1).

### B. Thermofluor indicates enhanced thermal stability of _Δ_TraG*_R100_

Thermofluor experiments were performed to compare the thermal stability of the TraG* variants. These assays involved the use of a hydrophobic fluorescent probe that is quenched upon addition to an aqueous sample of protein in a buffer.^54^ Heat is applied to the samples in a gradient fashion; as the protein begins to unfold it exposes hydrophobic residues that the probe binds to and fluoresces. The fluorescence signal is measured through spectrophotometry, as the heat continues to increase the protein begins to aggregate and hydrophobic residues become excluded from solvent, resulting in a loss of probe binding and a decrease in fluorescence. The produced profile is a melting curve that can allow for characterization of thermal stability through comparison of the melting temperature (T_m_), which is the midpoint of the unfolding transition, as determined through the highest value on the second derivative of the function. The assay can be altered to optimize buffer conditions to aid in protein stability, characterize the effect of mutations on the thermal stability of proteins, predict the crystallization propensity of proteins, and determine quantitative binding affinities between a protein and a ligand.^54–56^

**FIG. 3.**
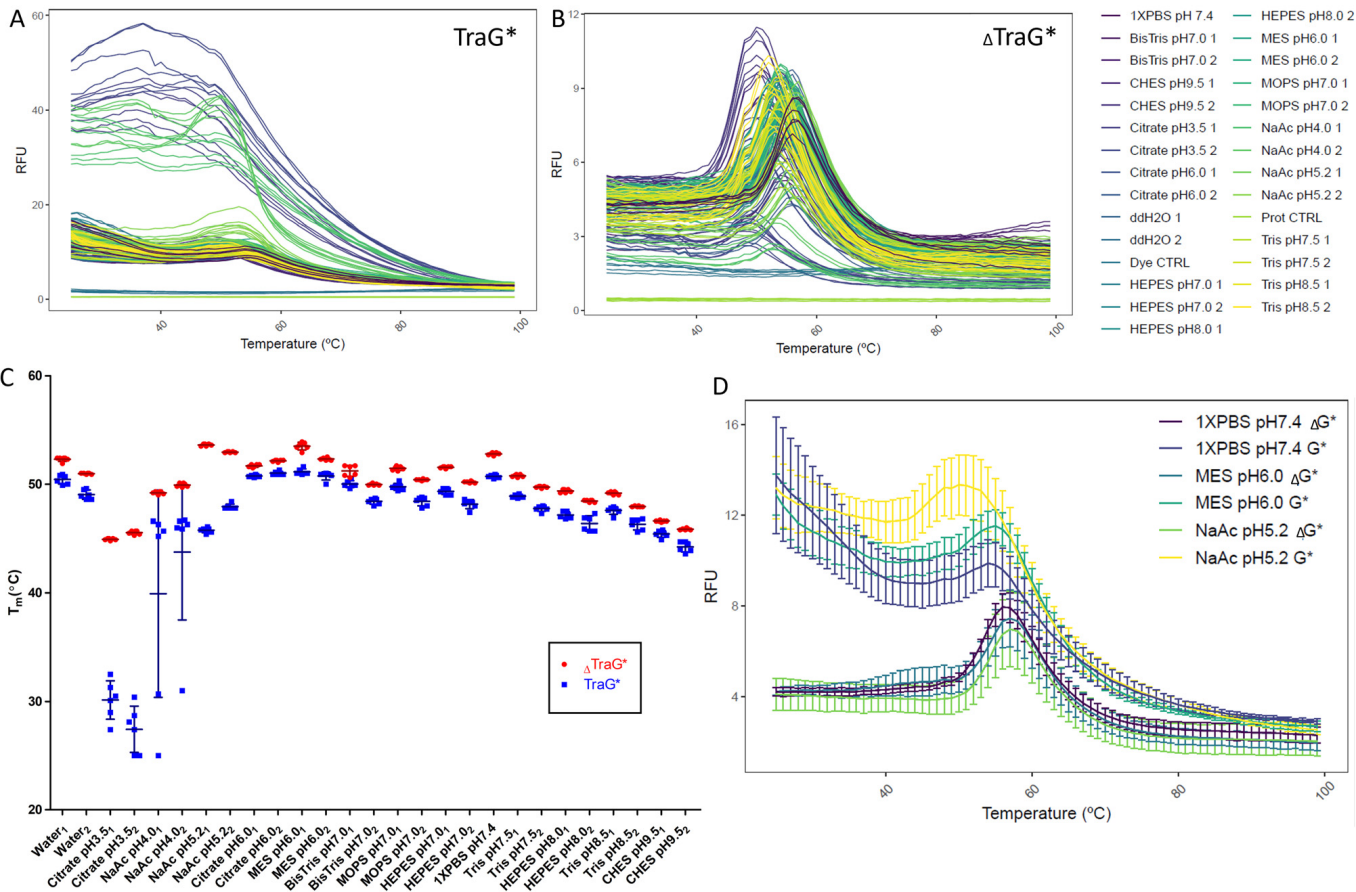
Melting curve plots displaying the temperature dependent unfolding of (a) HisTraG*_R100_ and (b) _Δ_TraG*_R100_ in various buffer conditions [seen in the legend right of (b)] as detected using SYPRO orange. Each buffer was at a concentration of 50 mM [except Nanopure water (ddH_2_O) and PBS], where one represents the addition of 50 mM NaCl in the buffer and two includes 200 mM NaCl. Six (6) replicates of each experiment are shown. (c) Mean melting temperatures with ±SD of the TraG* variants in all tested buffer conditions. (d) Average melting curve plots of the top three stabilizing buffer conditions for both _Δ_TraG*_R100_ and TraG*_R100_, as determined by the T_m_ of these average curves.

Thermofluor experiments demonstrated that _Δ_TraG*_R100_ is more stable than TraG*_R100_ based on lower average T_m_ values in 25 of the 27 tested buffer conditions (Fig. 3). In the remaining two conditions the error bars overlap, indicating there is no difference in their T_m_ when in the buffer. The difference in T_m_ between TraG*_R100_ constructs is minimal in many cases; however, the shapes of TraG*_R100_ melting curves are poorer than _Δ_TraG*_R100_, as the high fluorescence in the lower temperatures (25–45 °C) and the shallow peak indicates there are some TraG*_R100_ protein species present which have hydrophobic regions exposed immediately upon SYPRO addition [Fig. 3(a)].^43^ The variability in the TraG*_R100_ melting curves is higher than the truncated protein as demonstrated by the larger error, which may further indicate the instability of the protein as different batches produce protein samples with different thermal stability, while _Δ_TraG*_R100_ remains consistent between batches [Fig. 3(c)].^47^ Sodium acetate pH 5.2 and MES pH 6.0 were observed to be optimal buffers for TraG*_R100_ variants based on this assay [Fig. 3(d)]. As well, buffers with 50 mM NaCl appeared to have a more stabilizing effect than buffers with 200 mM NaCl.

### C. Improved chemical stability of _Δ_TraG*_R100_ as observed by circular dichroism

Circular dichroism (CD) is a widely used and well-developed technique for the determination of protein secondary structure.^51,57^ The technique relies upon the differential absorption of left- and right-hand circular polarized light. In this case, the electric field of a photon has a circularly rotational direction relative to the direction of propagation, where the photon vector remains constant in magnitude.^58,59^ When these photons pass through an asymmetric (chiral) molecule, the speed, absorbance and the wavelengths differ depending on the direction of their polarization. As the sum of the vectors for the right- and left- handed polarized light forms an ellipse, the change in ellipticity (measured as Δε) of a substance as a function of the wavelength of incident light is reported in performing CD experiments, although the change in absorbance of the differentially polarized light is what is measured in the CD spectrometer. As proteins are chiral molecules and secondary structures result in specific absorbance patterns at certain emission wavelengths, CD is widely used to determine the secondary structure content of proteins and can be used to determine the structural stability of proteins in solution.^58,60,61^

**FIG. 4.**
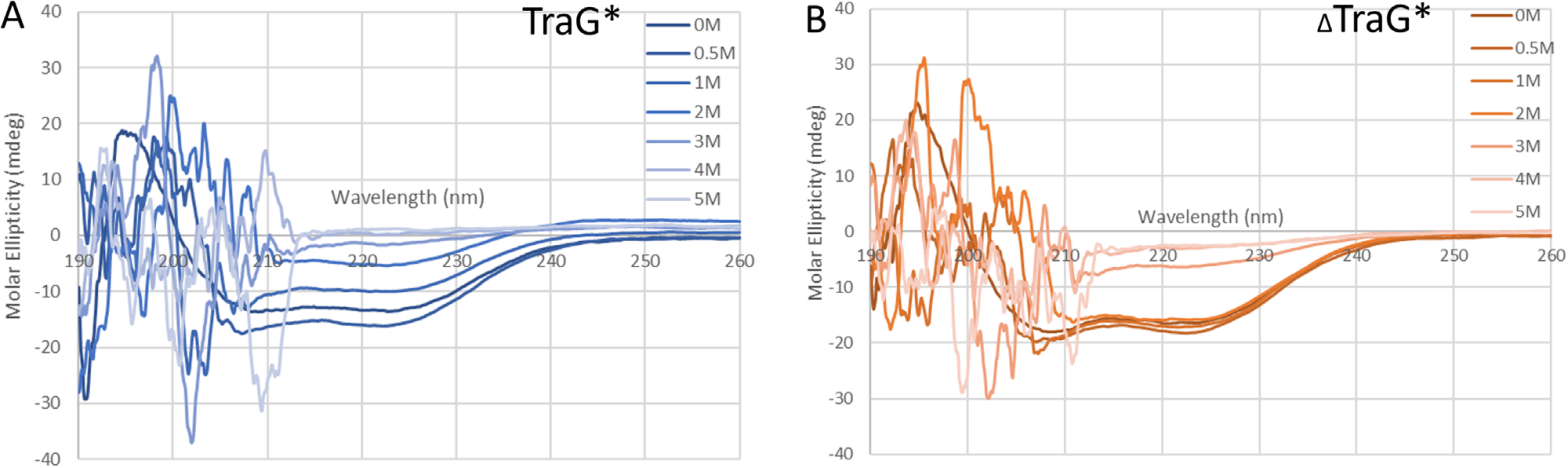
Urea denaturation of (a) TraG*_R100_ and (b) _Δ_TraG*_R100_ as measured by CD spectroscopy. All experiments were performed with a final protein concentration of 2 *μ*M and were incubated in the respective concentrations of urea (shown in the figure legends) for 1 h at 25 °C prior to UV measurement. The resultant data were normalized by subtracting using the blank spectra, wherein the buffer conditions in the same concentration of urea were replicated and CD data were collected. Molar ellipticity measurements were obtained every 0.1 nm from 260 to 190 nm. Data from 195–260 nm was entered into BeStSel for deconvolution,^51^ and the resultant analyses from triplicate runs is shown in Table II; spectra shown are from single representative experiments. Baseline spectra for both proteins are shown in supplementary material Fig. S2.

The CD spectra of both TraG*_R100_ variants appear to be mainly α-helical based on the characteristic negative Δε peaks at 222 and 208 nm (Fig. 4).^59^ Deconvolution in BeStSel (Table II) indicates differences in the extent to which the proteins fold into canonical α helices.^51^ The model providing the best fit for TraG*_R100_ indicated that 72.3% of the protein was predicted to be α-helical, albeit 20.9% of helices are of a bent or imperfect topology (supplementary material Fig. S2). Turns were of low abundance in the overall structure of the protein at 7.4%, and the remaining 20.3% of the protein was deemed as “other,” which include 3_10_ and π helices, but also loops that may not change the direction of polarized UV light and are therefore undetectable by CD.^51,62,63^ The spectrum of _Δ_TraG*_R100_ appears to have better defined peaks at 222 and 208 nm, and the protein is predicted to have 85.1% α-helices, with 67% of optimal topology contrasting with the 51.4% seen in TraG*_R100_. _Δ_TraG*_R100_ has only 14.8% other character and no strongly structured turns. This strongly indicates that the 45 residues removed from the protein are disordered; the flexible N-terminal region in TraG*_R100_ could be causing distortions in the remaining protein fold based on the predicted high relative change in the percentage of assigned secondary structure.

**TABLE II.** Secondary structure of TraG*_R100_ and ΔTraG*_R100_ under urea denaturation. Data presented from triplicate measurements and deconvoluted with BeStSel. Representative CD spectra shown in Fig. 4.

TraG*_R100_								
(Urea) (M)	α-Helix (%)		Antiparallel β-sheet (%)			Parallel β-sheet (%)	Turn (%)	Loop (%)
	Regular	Distorted	Left-twisted	Relaxed	Right-twisted			
0.0	40.70	17.10	0.00	2.97	0.00	0.00	8.47	30.77
0.5	18.37	16.80	0.00	0.00	14.73	1.03	9.10	40.03
1.0	19.13	19.07	6.97	3.60	12.33	0.00	12.37	26.57
2.0	8.50	9.00	5.07	2.60	9.90	6.70	11.27	47.00
3.0	5.07	11.17	2.33	0.00	12.43	0.00	15.63	53.37
4.0	0.00	0.00	3.50	21.13	22.80	0.00	19.90	32.70
5.0	8.10	10.80	3.40	5.93	8.80	0.00	15.20	47.77

ΔTraG*_R100_								
(Urea) (M)	α-Helix (%)		Antiparallel β-sheet (%)			Parallel β-sheet (%)	Turn (%)	Loop (%)
	Regular	Distorted	Left-twisted	Relaxed	Right-twisted			
0.0	64.20	17.13	0.00	0.03	0.03	1.47	6.77	10.30
0.5	34.63	16.57	0.00	0.00	26.83	0.87	7.07	14.10
1.0	28.10	19.40	9.03	0.00	7.23	0.77	8.77	26.70
2.0	24.43	10.80	10.20	0.00	8.57	10.03	5.87	30.17
3.0	7.50	8.27	0.00	6.77	17.77	0.00	18.40	41.33
4.0	9.37	0.00	0.00	15.50	12.27	3.57	20.67	38.63
5.0	1.37	9.00	0.90	3.30	5.90	0.00	18.83	58.63

Urea denaturation studies were performed to support the conclusion surmised from the Thermofluor data that _Δ_TraG*_R100_ has higher stability than TraG*_R100_. In comparing spectra and tables displaying BeStSel^51^ predicted secondary structure topology of TraG*_R100_ to _Δ_TraG*_R100_, it is evident that the presence of residues A452-A496 destabilizes TraG*_R100_ (Fig. 4, Table II). TraG*_R100_ displayed higher susceptibility to unfolding based on the direct decrement of Δε at 222 and 208 nm as the urea concentration was increased. The secondary structure of _Δ_TraG*_R100_ remained relatively intact until incubation with 3.0M urea, while the secondary structure of TraG*_R100_ was partially unfolded at 1.0M urea based on the topology analysis from 260 to 200 nm by BeStSel (Table II).^51^

### D. CIU–MS demonstrates differences in conformational stability of TraG*_R100_ and _Δ_TraG*_R100_

Collision-induced unfolding (CIU) mass spectrometry is a reliable technique for studying and comparing a protein's conformational stability.^64–67^ CIU–MS employs an ion mobility spectrometry (IMS) cell within the mass spectrometer that is filled with an inert gas and provides a weak electric field gradient that filters ionized species separated in the previous trap cell. Drift time in the IMS cell, which is defined by the time an ion takes to travel the distance from the drift tube to the detector, is dependent on mass and size as well as the conformational shape of the fragment. In CIU–MS, the trap collision energy (CE) is increased in a stepwise fashion, allowing for breakages of non-covalent bonds that can be visualized by IMS as a shift to a slower moving, unfolded species. Therefore, CIU provides an indication of the conformational stability of proteins; a protein that has a lower number of drift time distributions (representing different conformations) and/or remains in a lower drift time distribution at a higher CE has higher conformational stability. The native mass spectra of TraG*_R100_ and _Δ_TraG*_R100_ were highly reproducible in biological replicates, where TraG*_R100_ had highly abundant charge states of 18+ (3144 m/z), 17+ (3329 m/z), and 16+ (3537 m/z) while _Δ_TraG*_R100_ had more abundant 17+ (3054 m/z), 16+ (3245 m/z), and 15+ (3462 m/z) charge states (supplementary material Fig. 3). The propensity for TraG*_R100_ to occupy higher energy charge states more frequently indicates that the presence of the putative linker region results in lower stability.^68^ The mass of each protein as determined using ESIprot was 56 588 ± 8.71 Da for TraG*_R100_ and 51 906 ± 7.54 Da for _Δ_TraG*_R100_.^69^

**FIG. 5.**
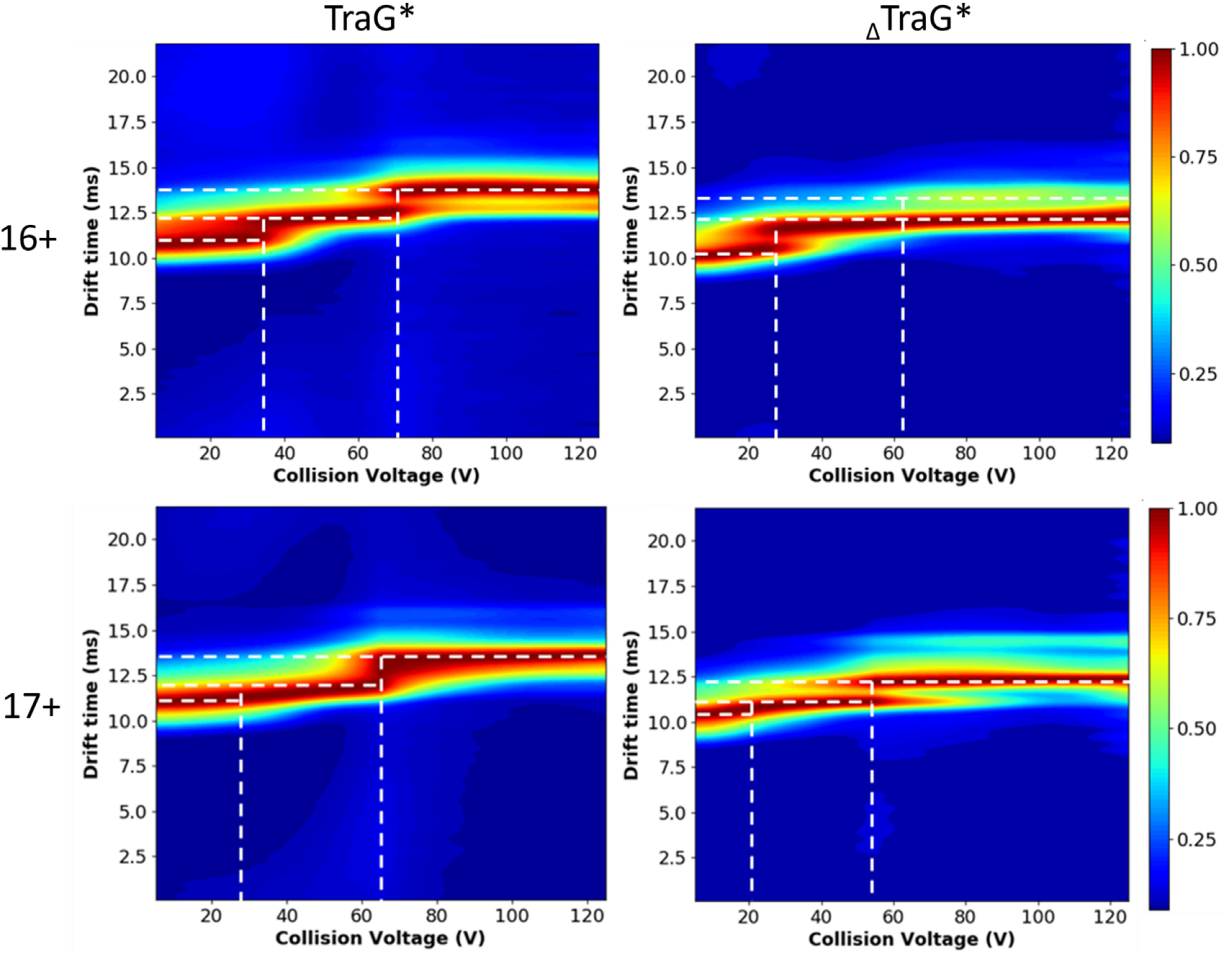
CIU–MS heat maps displaying the drift time of the 16+ and 17+ charge states of TraG*_R100_ and _Δ_TraG*_R100_ as trap collision energy is increased from 5 to 125 V. These maps were plotted with CIUSuite2^70^ and smoothed with default settings from the software. The m/z range set for plotting the IMS of the 17+ charge states were 3320–3355 for TraG* and 3040–3090 for _Δ_TraG*, and the m/z range used for the 16+ charge states were 3525–3575 for TraG* and 3230–3280 for _Δ_TraG*. A biological replicate for each experiment is shown in supplementary material Fig. S5.

CIU data were analyzed using charge states 17+ and 16+ for both proteins, as the 15+ and 18+ charge states were of insufficient abundance in some CEs to be compared. To visualize changes in drift time of these species as a function of CE, CIUSuite2^70^ was used to generate CIU heat maps (Fig. 5). The replicates of each plot have some differences in the intensity of the transitions; however, there are common shifts in drift time for the same species (supplementary material Fig. S5). The 16+ charge state of TraG*_R100_ displays two shifts, one at ∼35 V CE and one at ∼70 V CE. The 16+ charge state of _Δ_TraG*_R100_ has a single shift at ∼25 V CE; however, there appears to be a low abundance of an unfolded state from ∼60 V CE onward. Although _Δ_TraG*_R100_ may transition to a conformational state with slightly less energy, the lower abundance of the species with the longest drift time seen in TraG*_R100_ indicates a significantly unfolded conformation is achieved more commonly when the putative linker region is present. A similar trend is seen in the 17+ charge state as well. This can also be visualized by observing the trends in the IMS spectra, which are more reproducible and comparable between replicates (supplementary material Fig. 4).

### E. SEC–MALS–SAXS aids in defining _Δ_TraG*_R100_ monomer shape

Size exclusion chromatography linked to multi-angled light scattering and small angle x-ray scattering (SEC–MALS–SAXS) is an equilibrium based SAXS method, which links multiple biophysical techniques to accurately inform on particle shape and size.^71^ SAXS involves the illumination of an aqueous sample by a collimated monochromatic x-ray beam, where the particles in solution will scatter the x rays.^72^ The intensity of this scattering is detected; then, the scattering of the solvent without the particles of interest is determined and subtracted to provide the scattering pattern of the particles of interest. This is simplified through the inclusion of SEC as particles will be separated by size, and highly accurate solvent frames can be collected prior to the void volume and after the total column volume.^71^ Coupling SEC –SAXS with MALS, which is the use of monochromatic incident light to cause Rayleigh scattering that is detected upon 17 angles, and a direct refractive index (dRI) detector, which measures refractive indices of a solution in a flow cell and the solvent in a reference cell to determine the RI produced from the particles of interest, has become established as a robust method for acquiring highly accurate biophysical data.^71^ MALS and dRI detectors provide a more accurate molecular weight determination than SAXS and provide radius of hydration R_h_ values (the radius of the hypothetical sphere of aqueous solvent that diffuses at the same rate as the macromolecule) to couple with radius of gyration R_g_ (the radius obtained from the rotationally averaged volume of the macromolecule) values obtained from SAXS.^71–73^

**FIG. 6.**
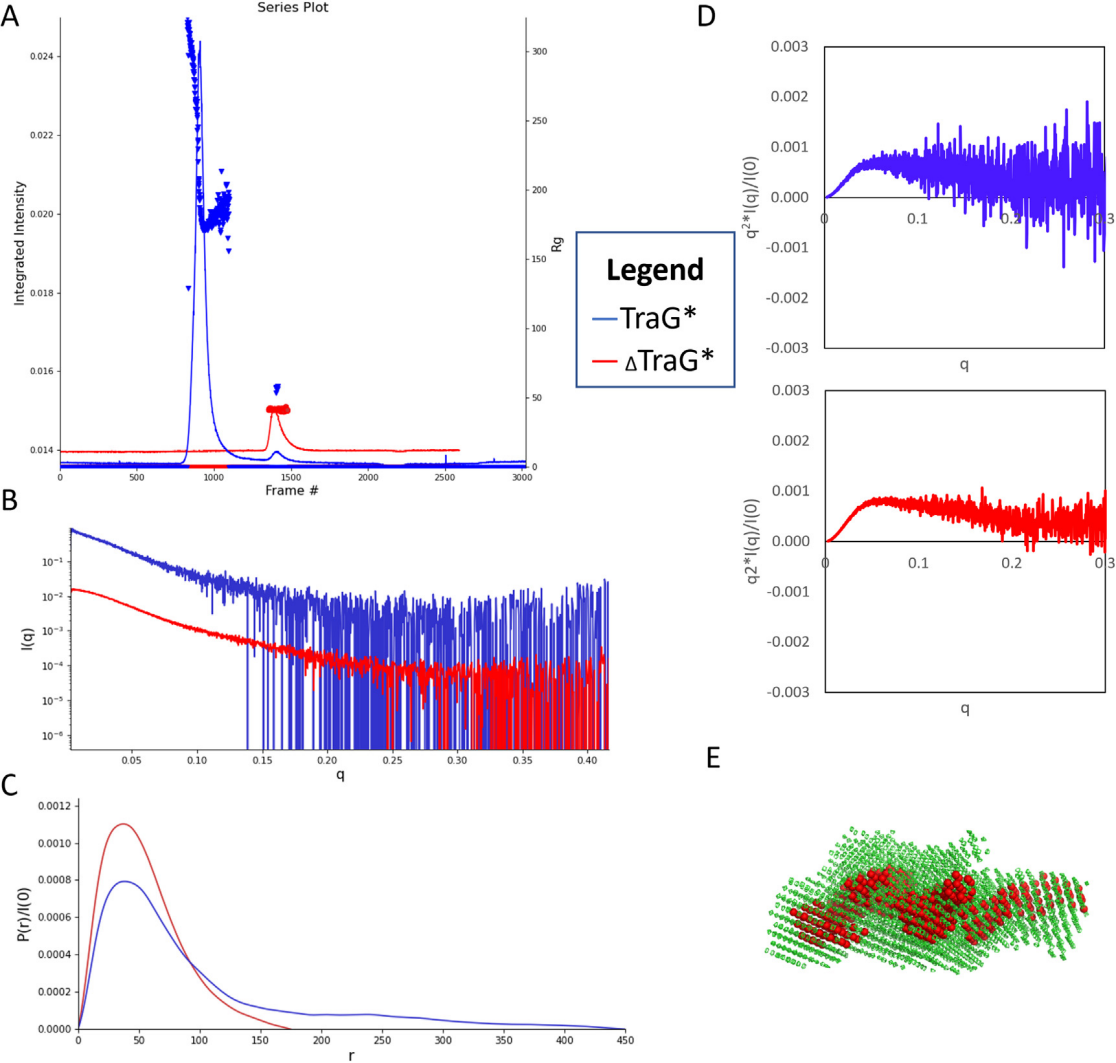
(a) SEC –MALS–SAXS chromatogram displaying the integrated intensity of diffraction and radius of gyration (R_g_) resulting from each frame of data collected for TraG*_R100_ (blue) and _Δ_TraG*_R100_ (red). (b) Scattering profiles of TraG*_R100_ (blue) and _Δ_TraG*_R100_ (red) show the abundance of noise in the TraG*_R100_ profile and noise in the high q regions for _Δ_TraG*_R100_. (c) P(r) functions of TraG*_R100_ (blue) and _Δ_TraG*_R100_ (red) were produced using GNOM(IFT) and optimized for R_g_ values comparable to those resulting from Guinier analysis; Dmax of TraG* was truncated at 450 Å while _Δ_TraG* was cut at 175 Å to allow for some. (d) Normalized Kratky plots resulting from the scattering profile of TraG*_R100_ (blue) and _Δ_TraG*_R100_ (red). (e) A bead model of _Δ_TraG*_R100_ which displays the smallest shell (red) with mesh models (green) of the highest shell from DAMCLUST refinement, produced from the P(r) function. This reconstruction had an ambiguity score of 1.940 with 87 ambiguity categories based on AMBIMETER. All SAXS data images were produced using RAW, and bead models were visualized using Pymol.^52,103^

SEC–MALS–SAXS data of 5 mg/ml TraG*_R100_ indicate that it had aggregated prior to experimentation [Fig. 6(a)]. A highly intense peak at frames 700–1100 is confirmed to result from high molecular weight aggregates of the protein as it was eluted in the SEC column's void volume. The low intensity of the monomeric protein peak at frames 1400–1480 results from loss of monomeric TraG* _R100_ due to aggregate formation. This is also seen in the MALS data by the large light scattering peak at 10.5–12.5 ml and the lower light scattering from the UV_280 nm_ and the dRI peak seen in 20.5–21.5 min of elution (supplementary material Fig. S6). The resultant SAXS data were buffer subtracted using frames 315–400 and the sample region was chosen as frames 928–939. Evolving Factor Analysis (EFA) was performed on the region 1300–1520 to separate the peak associated with the aggregate from the monomeric protein peak. Two (2) significant singular values were used, with component ranges from frames 1300–1481 and 1340–1520, with the latter range plotted as the scattering profile for further analysis [Fig. 6(b)]. **_Δ_**TraG*_R100_ at 5 mg/ml shows the presence of monomeric protein at frames 1409–1460, with no apparent aggregation seen [Fig. 6(a)]. The buffer region used for subtraction was 980–1157, and a linear baseline correction was performed using frames 26–70 and 2550–2594.

Guinier analysis (a function which allows for observation of scattering at the smallest q values) of the SAXS scattering profiles seen in Fig. 6(b) results in R_g_ and correlated molecular weight values of 57.68 ± 0.73 Å and 76.6 kDa, 41.27 ± 0.18 Å and 56.4 kDa for TraG*_R100_ and **_Δ_**TraG*_R100_, respectively (Guinier analyses seen in supplementary material Fig. S7). The R_g_ of **_Δ_**TraG*_R100_, as calculated from the pair-distance distribution P(r) function from the GNOM program,^52,74^ was reasonably close to the R_g_ from Guinier analysis at 44.14 ± 0.23 Å. However, the R_g_ of TraG*_R100_ from the P(r) function was 91.23 ± 3.40 Å and the maximum dimension Dmax [the largest paired-distance achieved between points on the molecule as determined by the P(r) function]^72^ was 450 Å, which is highly overestimated and likely due to the presence of residual aggregated protein [Fig. 6(c)]. In SAXS, the P(r) function is used to describe the distances between all scattering points on the macromolecule, grouped into paired-sets, in order to predict the overall shape of the molecule.^75^ In comparing the molecular weight estimates from the Guinier analyses to the estimates acquired from MALS data, the estimate for TraG*_R100_ is proximal to the MW from the Guinier analysis at 75 710 ± 53.83 Da; however, the MW estimate for **_Δ_**TraG*_R100_ is lower at 50 650 ± 1.06 Da, which is closer to the molecular weight determined from MS (51 906 ± 7.54 Da). The R_h_ values, as obtained from MALS, were 67.7 ± 0.014 and 47.8 ± 0.007 Å for TraG*_R100_ and **_Δ_**TraG*_R100_, respectively.

Kratky plots are used to qualitatively assess the flexibility and/or the degree of unfolding in samples and are mathematically expressed as q^2^I(q) vs q.^72,76,77^ The shape of a Kratky plot dictates the protein's folding, globular proteins will have a Gaussian peak, unfolded proteins will have a plateau at high q, while prolate or partially unfolded proteins will have a combination of bell-shape and plateau. A normalized Kratky plot normalizes scattering profiles by mass and concentration by plotting q^2^I(q)/I(0) vs q. A normalized Kratky analysis was performed on both scattering profiles, and the **_Δ_**TraG*_R100_ curve had a slight bell shape returning to the x-axis with higher q values, indicating some disorder is present [Fig. 6(d)]. The normalized Kratky plot for TraG*_R100_ was highly unfolded based on the lack of a Gaussian peak; however, this qualitative analysis is obscured by significant noise at high q, likely due to the low concentration of the monomeric protein. The Guinier analysis of TraG*_R100_ also showed that the monomeric peak has some residual aggregate, as the scattering profile displayed curvature at low q data (supplementary material Fig. S7). The Guinier analysis showed some minor curvature in low q data for **_Δ_**TraG*_R100_ (supplementary material Fig. S7) and the scattering profile showed some noise in high q data [Fig. 6(b)]. However, this could not be truncated as the shape of the P(r) function was desirable at a Dmax of 175 Å wherein the curve smoothly approaches the x-axis [Fig. 6(c)]. AMBIMETER was used to determine ambiguity of the potential output models; the **_Δ_**TraG*_R100_ model was reasonable with an ambiguity score of 1.940 and 87 ambiguity categories, while the TraG*_R100_ model was ambiguous as it had an ambiguity score of 0 with 0 compatible shape categories [Fig. 6(e)].^78^ The TraG*_R100_ model is not shown as it is ambiguous; however, it can be viewed on the SASBDB (https://www.sasbdb.org) under accession code SASDQH6.^104^ The produced **_Δ_**TraG*_R100_ bead model appears to be a coiled rod-like shape (SASDQG6),^104^ whereas the TraG*_R100_ model is a less-defined and further extended structure. The models cannot be accurately compared as the additional lobes seen in the TraG*_R100_ bead model may result from disruption by the scattering of the residual aggregate protein rather than from the movement of a flexible N-terminal domain.

## IV. DISCUSSION

TraG is a multifunctional protein in T4SS encoded on F-like plasmids, including the R100 plasmid. It is composed of a membrane-bound N-terminal domain required for pilus generation and a C-terminal periplasmic domain (TraG*) required for Eex and Mps. TraG* is logically presumed to have some plasticity in its structure to facilitate these different functions that are expected to require numerous protein–protein interactions (PPIs). Based on the findings presented, it can be stated that the N-terminal region of TraG* from the R100 plasmid (aa A452-A496 of TraG_R100_) is highly dynamic and its presence destabilizes the protein. The characteristics of higher thermal and chemical stability of the truncation mutant observed from Thermofluor and urea denaturation CD experiments are sufficient in stating that _Δ_TraG*_R100_ is more stable than TraG*_R100_. In addition, the reduced unfolded population observed in CIU–MS for _Δ_TraG*_R100_ provides further evidence that the removal of the N-terminal region stabilizes the protein. However, it is not sufficient in identifying the deleted region as an intrinsically disordered region (IDR). IDRs shorter than 50 residues are not uncommon, however they typically serve a functional purpose in the native protein.^79^ As this region has not been shown conclusively to be intrinsically disordered in full-length TraG_R100_, and its function is not yet confirmed, the region cannot be classified as an IDR. However, the TraG*_R100_ constructs explored herein provide evidence that the presence of the region from residues A452-A496 results in a protein which has a higher prevalence for aggregation as shown in SEC –MALS–SAXS (Fig. 6). The region likely serves as a flexible linker in the full-length protein; however, when expressed separately from the N-terminal membrane bound portion, this region of TraG*_R100_ displays higher dynamicity due to its expression as a synthetic construct.

AlphaFold and RoseTTAFold both predict the linker region of TraG to contain a pseudo β-sheet surrounded by loop regions [Fig. 2(f)]. This region may fold cryptically and uncoil like a spring to extend the periplasmic TraG* to its interacting partner TraS in the IM of the recipient cell. While AlphaFold and RoseTTAFold predictions provide evidence of a flexible N-terminal linker region in TraG*, they also indicate that a C-terminal deletion may be required as well to make the protein more amenable to crystallization (Fig. 2). Based on the models which predict the protein as mainly consisting of extended α-helices, it is visualized why TraG*_R100_ aggregates excessively rather than crystallizing; non-covalent interactions promoted by crystallization conditions may induce the formation of nonspecific coiled-coils.^80^ Additionally, native mass spectra show a large proportion of unstructured species in both TraG*_R100_ and **_Δ_**TraG*_R100_ (supplementary material Fig. S3), demonstrating the dynamic nature of the protein regardless of the N-terminal truncation. Another insight from the artificial intelligence (AI)-predicted structures is the region of TraG* known to interact with TraS is predicted to be an extended loop. This topology could be required for its functionality wherein this region may become structured only when interacting with its binding partner. Determining the crystal structure of _Δ_TraG*_R100_ that includes this interacting motif will shed light on the role of this extended loop in TraG–TraS interactions; these studies are ongoing.)

As the only deletion mutant tested was from the truncation of 45 amino acids, the size of the disordered region in TraG*_R100_ has not been defined in full; however, the findings of this study have several implications. Many structural predictive software packages such as Phyre2, IUPred2A, ANCHOR2A, and AlphaFold^40,81–83^ improperly identified secondary structure properties of TraG*, overestimating the disordered content of the periplasmic protein to be approximately 50%.^40,81–83^ CD analysis of TraG*_R100_ (supplementary material Fig. S2) indicates the protein is not predominantly disordered, rather some regions are predicted to feature distorted helices, at 20.3% of the protein. This indicates that these programs are incorrectly assigning regions of TraG*_R100_ as disordered, likely due to an inability to identify homologs with known structures, enforcing the postulation regarding the novelty of this protein structure. The identification of a highly dynamic region by the PSP software^39^ suggests that other popular algorithms should be modified to include disorder predictions based on long range pi–pi contact frequencies to improve their accuracy.

The increased thermal stability of **_Δ_**TraG*_R100_ was demonstrated quantitatively via the Thermofluor assays (Fig. 3). The mean T_m_ of **_Δ_**TraG*_R100_ in all buffers tested (excluding 50 mM citrate pH 3.5) was 50.6 °C, while the mean T_m_ of TraG*_R100_ was 48.0 °C. The difference in T_m_ is not the only factor that changes when TraG*_R100_ is truncated, the shape of the melting curves is improved as the relative fluorescence units (RFUs) at initial temperatures is lowered, indicating a reduced quantity of **_Δ_**TraG*_R100_ molecules are unfolded at the beginning of the assay relative to TraG*_R100_, and the peak RFU at the T_m_ is further pronounced in most of the conditions attempted. The large values of the standard error in many of the TraG*_R100_ experiments are likely due to differences in abundance of aggregation of the protein prior to the start of the experiment; the thermal stability of the truncation mutant is consistent between replicates as the error bars for all buffers tested is smaller, indicating less batch-to-batch variability. Although **_Δ_**TraG*_R100_ has higher thermal stability relative to TraG*_R100_, the mean T_m_ of the lysozyme control is 69.8 °C, indicating that **_Δ_**TraG*_R100_ is not as stable as a small, well-folded globular protein.

The increased T_m_ of conditions where buffer pH was approximate to the respective predicted isoelectric point (pI) of the proteins (from ProtParam,^84^ pI TraG*_R100_: 5.83 and pI _Δ_TraG*_R100_: 5.95) is an interesting phenomenon. Maintaining protein charge at net neutral is predicted to aid in the formation of intermolecular interactions, which may be the reason for increased thermal stability seen in these conditions. Protein crystallization is often suggested to be started with the pH of the mother liquor equal or proximal to the pI of the protein,^85,86^ suggesting optimal buffers of sodium acetate (pH 5.2) and MES (pH 6.0) be explored. Buffers with 50 mM NaCl concentrations appeared to better maintain folding of both proteins as temperature was increased in comparison to buffers with 200 mM NaCl. This could be caused by an increased heat capacity of the solvent when less ionic strength is present, or from higher ionicity affecting intermolecular interactions by masking charges in TraG*_R100_, or that both phenomena contribute to the observed changes. As the thermocycler employed for the Thermofluor assay measures the internal temperature of the instrument rather than the temperature of the buffers themselves, it is possible the buffers with higher salt content absorbed more kinetic energy at the same temperatures and therefore caused the proteins to unfold at slightly lower temperatures. Additionally, increasing salt concentrations may create “salting out” effects; as ionicity is increased salt ions can mask the inter- and intra- molecular ionic interactions that prevent protein unfolding at increased temperatures.^87^

Circular dichroism revealed the changes in secondary structure that occur when the 45 N-terminal residues of TraG*_R100_ are removed. The increase in definition of characteristic absorbance peaks for α-helices at 222 and 208 nm wavelengths is highly indicative of an increase in α-helical character by the truncation of the flexible region. This is quantified by BeStSel,^51^ which predicts an increase in the α-helical character and a decrease in loop character in the absence of the N-terminal region (supplementary material Fig. S2). The predicted change in α-helical topology of 15.6% is higher than expected for a loss of only 8.6% of the protein's residues, thus indicating the presence of the flexible region disrupts the topology of the remaining protein. This provides a biophysical explanation for the observed aggregation propensity for TraG*_R100_ relative to _Δ_TraG*_R100_ and explains difficulties in attempts to crystallize TraG*_R100_. The high dynamicity of TraG_R100_ residues A452-A496 when not anchored to the C-terminal intermembrane domain results in changes to the overall topology of TraG*_R100_.

Urea denaturation revealed that _Δ_TraG*_R100_ has higher chemical stability than TraG*_R100_, as observed by the loss of characteristic α-helical absorbance at lower urea concentrations. For TraG*_R100_ at 1 M urea, the absorbance peaks of 222 and 208 nm were significantly less pronounced, almost decreasing by 50% of their values from the native spectra (Fig. 4). The truncation mutant shows only a minor change in spectra from incubation with 1 M urea in the respective α-helical regions. Quantification via BeStSel predictions indicated a 19.13% α-helical character after incubating TraG* _R100_ in 1 M urea while _Δ_TraG*_R100_ had 28.10%, both from an average of three replicates (Table II). Together with the Thermofluor data demonstrating the lowered thermal stability of TraG*_R100_, the urea denaturation and native CD experiments demonstrate an increase in overall stability of the truncation mutant _Δ_TraG*_R100_.

Native ESI–MS confirmed the masses of the proteins. The changes relative to expectations based on sequence (TraG*_R100_: 56 699.30 Da, _Δ_TraG*_R100_: 52 010.34 Da via ProtParam^84^) might be the result of salt adducts carried over from the purification and potential degradation from the ESI process (supplementary material Fig. S3). The spectra show that both TraG*_R100_ and _Δ_TraG*_R100_ samples had unfolded conformations as seen in the 2000–2700 m/z range. However, the peaks seen in the TraG*_R100_ spectrum are more abundant indicating a higher propensity for unfolding in TraG*_R100_. The spectra also demonstrate that TraG*_R100_ has an increased propensity for occupying higher energy charge states of 18+ and 17+ relative to _Δ_TraG*_R100_, further indicating the lowered conformational stability of TraG*_R100_.^88^

CIU-MS of the TraG*_R100_ constructs further identifies the lowered conformational stability of TraG*_R100_. The third TraG*_R100_ species with the longest drift time (∼14 ms) is observed at nearly 100% abundance for both the 16+ and 17+ charge states above 70 V CE, whereas for _Δ_TraG*_R100_ the third species (∼13.5 ms) is only partially occupied above 70 V CE and not well defined for the 17+ charge state (Fig. 5). This indicates a third, highly unfolded conformer is more often induced in TraG*_R100_ than _Δ_TraG*_R100_, supporting the interpretation that the flexibility of the N-terminal region affects the conformational dynamics of TraG*_R100_. The consistency between biological replicates is evident when viewing the IMS spectra (supplementary material Fig. S4). _Δ_TraG*_R100_ and TraG*_R100_ have three conformers each: approximately 10.5, 12.5, 13.5 and 11, 13, 14 ms, respectively, occupied in differing percentages depending on the CE applied. The differences in drift time between conformers of TraG*_R100_ and _Δ_TraG*_R100_ are appropriate based on a 45-residue truncation. The shift to the 14 ms conformer is well defined for TraG*_R100_ in the 16+ charge state at 100 V CE; however, the IMS spectrum of the 17+ charge state has a broad peak at 100 V CE that is centered at a smaller drift time (∼13.75 ms) when compared to the drift time for the most unfolded conformer of _Δ_TraG* (∼14.5 ms). Although there is an unfolded conformer for _Δ_TraG*_R100_ with a longer drift time (∼14.5 ms), it is of low abundance at ∼40% signal, whereas the second conformer (∼12.5 ms) is at 100% abundance, and the most folded conformer is present at ∼30% signal (∼10.5 ms). Therefore, the interpretation that TraG*_R100_ can access a more unfolded conformational state at a higher propensity than _Δ_TraG*_R100_ is maintained. However, as each charge state was not isolated during data collection prior to the CIU analysis, charge stripping events may have caused some additional noise in the data.^66,67^ Therefore, no quantitative claims can be made from this CIU MS data regarding the degree of disorder in each protein construct.

The N-terminal residues of TraG*_R100_ greatly increase the tendency of the protein to aggregate as was demonstrated via SEC–MALS–SAXS (Fig. 6). The high MW peak representing MDa-sized complexes of aggregates is present in TraG*_R100_ despite the addition of the detergent NP40 to aid in maintaining solubility of the protein. This aggregate was not detectable in the sample of _Δ_TraG*_R100_ by SAXS or MALS, furthering the interpretation that the protein's stability is greatly increased by the truncation performed in this study. The SEC–MALS–SAXS data from _Δ_TraG*_R100_ provided a good MW determination from MALS, at 50 650 Da it was close to the weight provided from MS (51 906 Da) and the estimated weight from ProtParam (52 010.34 Da). SAXS data overestimated the MW as 57.68 kDa and provided a reasonable R_g_ determination from Guinier analysis (41.27 Å) that was proximal to the R_g_ from the P(r) function (44.14 Å) by ∼3 Å. The R_h_ determination from MALS provided a value of 47.8 Å, which allows for a shape factor calculation based on the R_g_/R_h_ ratio, resulting in a value of 0.86–0.92 for _Δ_TraG*_R100_.^71^ This represents a more elongated structure as shape factors of ∼0.75 are very compact, globular proteins and become more prolate as the shape factor increases. Additionally, the low peak of the bell-shaped Kratky plot for _Δ_TraG*_R100_ [Fig. 6(d)] indicates that the protein has some unstructured properties, which may be the reason for some noise at high q in the Guinier analysis (supplementary material Fig. S7) and the ambiguity score of 1.940 for the P(r) function by AMBIMETER.^52,78^ Based on the software structural predictions and the known function of TraG*_R100_, it is reasonable to conclude the overall structure of the protein would require flexibility and, therefore, have some intrinsically disordered properties. In comparing the produced bead model of _Δ_TraG*_R100_ [Fig. 6(e)] to the AlphaFold and RoseTTAFold predicted structures (supplementary material Fig. S1), an elongated structure is confirmed to be present and TraG*_R100_ is highly likely to consist of extended α-helices as predicted. The prolate structure of _Δ_TraG*_R100_ is also supported by the biochemical data shown in this study and is likely of significance for its function in extending to contact its protein partners. Due to the disruptive aggregation in the TraG*_R100_ samples, MW analyses, R_g_ analyses and bead model reconstructions resulting from SEC –MALS–SAXS are difficult to interpret. It may be true that the bead model of TraG*_R100_ would have a further extended structure due to the addition of the flexible N-terminus; however, due to the overestimated R_g_ and D_max_ the reconstruction cannot be compared to _Δ_TraG*_R100_ to describe how the addition of the N-terminal region affects these values and the low-resolution structural model.

## V. CONCLUSIONS

The findings presented here imply that the N-terminal region of TraG*_R100_ is highly flexible and results in the destabilization of the protein, resulting in lowered thermal and chemical stability, increased conformational dynamics and a higher aggregation propensity, thus making it difficult to characterize structurally. This was determined through comparisons of TraG*_R100_ with an N-terminal truncation mutant _Δ_TraG*_R100_, which presents a more stable protein. This supports the conclusion that the N-terminal region of TraG*_R100_ acts as a flexible linker that connects the membrane-bound portion of TraG_R100_ and periplasmic TraG*_R100_. This is congruent with known moieties in structural biology as the occurrence of unstructured regions approximately 50 residues in length is common in functional proteins.^89–91^ Intrinsically disordered proteins (IDPs) are more common in eukaryotes and viruses due to the requirement of complexity in their morphologies; it is suggested that there exists a link between intrinsic disorder and evolution.^92^ There are many examples of prokaryotic IDPs however, such as the proteins that regulate the assembly of large multiprotein complexes such as FlgE in the bacterial flagellum,^93^ and Ffh and FtsY of the ribosome.^89,94,95^ In both cases there exists an intrinsically disordered region (IDR) that serves as a linker region for two independent functional domains required in achieving a variety of conformations for important protein–protein interactions (PPIs). As well, several bacterial regulatory proteins have conserved short intrinsically disordered linker regions called Q-linkers.^89,96,97^ Therefore, it is not uncommon in bacterial proteins for disordered regions to be important flexible linkers of folded domains. It is possible this region is merely a linker and does not contain interacting domains, making it non-essential in the protein's structural solution. However, this is difficult to assess *in vivo* as experiments such as conjugative mating assays to test this theory are not possible as TraG* is nonfunctional when expressed on its own; biological function requires full-length TraG.^32^

TraG and TraS form the Eex system of the F-like T4SS and are heavily relied upon for preventing donor-donor plasmid exchange.^98,99^ As excessive conjugation can lead to lethal zygosis, it has been theorized that disruption of Eex systems would be detrimental to bacterial colony survival.^100^ Development of novel mechanisms to disrupt TraG–TraS interactions and cause ceaseless conjugation or completely prohibit conjugation is contingent on the structural knowledge of the system's protein subunits. Therefore, it is important to understand the structural characteristics of TraG and TraS, and how they interact within the Eex context. TraG*_R100_ and TraG*_F_ are predicted to be highly similar in structure based on overall sequence similarity; however, the region predicted to interact with TraS exhibits more sequence variation that the rest of the protein, which in turn provides plasmid-specific Eex.^32^ This study indicates that TraG*_R100_ is a structurally elongated protein with a highly dynamic N-terminal region that likely plays important functional roles in mediating interactions involved in Eex (with TraS) and Mps (with TraN) within the F-like T4SS conjugative assembly.

## Data Availability

The data that support the findings of this study are available from the corresponding author upon reasonable request. SAXS data for TraG* from the R100 plasmid are available in SASBDB (https://www.sasbdb.org) under accession codes SASDQG6 and SASDQH6.^104^
